# Preliminary Investigation into the regeneration of luminescent signal in nanoDot OSLDs

**DOI:** 10.1002/acm2.13035

**Published:** 2020-10-01

**Authors:** Kevin Liu

**Affiliations:** ^1^ Columbia University New York NY USA

**Keywords:** deep traps, optically stimulated luminescence dosimeter, radiation detectors, x rays

## Abstract

**Purpose:**

Reuse of optically stimulated luminescent dosimeters (OSLDs) has been suggested in prior works by using a light source to erase the dosimeter’s signal (optical bleaching) and recharacterizing the dosimeter’s sensitivity based on its dose history. However, depending on the wavelength of the bleaching source and the dosimeter’s dose history this may be problematic due to the presence of deep dosimetric traps and the phototransfer mechanism observed in Al_2_O_3_. In this work we examine the regeneration of signal in OSL nanoDots, with prior irradiation history, following their bleaching from a light source containing blue wavelengths.

**Methods:**

Irradiations were performed on 33 nanoDots at a dose range of 5–3000 cGy using 6 MV and 1000–3000 cGy using 220 kV x rays, with three nanoDots irradiated at each dose value. Following their irradiation, nanoDots were bleached using blue light for a period of 1 h. The postbleached signal in nanoDots was measured over a 27‐day period to track any changes in their measured signal due to the migration of charge carriers from deep dosimetric traps to shallower traps of the dosimeter.

**Results:**

The growth extent and growth rate observed in bleached nanoDots were observed to be dependent on the dosimeter’s accumulated dose history and energy of the radiation source. The 12 nanoDots with prior irradiation history of 500, 1000, 2000, and 3000 cGy from 6 MV x rays exhibit an increase in measured signal that range from 220 to 5229 PMT counts when measured the first day postbleaching to 408–8710 PMT counts when measured on the 27th day postbleaching, which may be substantial depending on the application regarding the dosimeter's reuse.

**Conclusion:**

These findings caution against the reuse and optical bleaching of nanoDots with prior irradiation history exceeding 100 cGy and demonstrate an energy, accumulated dose, and time dependence in the regeneration of signal in postbleached nanoDots with prior irradiation history.

## 1 INTRODUCTION

Optically stimulated luminescent dosimeters (OSLDs) are radiation detectors used to measure the absorbed dose at a point from exposure to ionizing radiation. OSL nanoDots are a type of OSLD that consist of a 1 cm × 1 cm × 0.2 cm plastic case containing a 4‐mm‐diameter disk of aluminum oxide doped with carbon (Al_2_O_3_:C). When Al_2_O_3_:C is exposed to ionizing radiation, free electrons and holes are generated whereby electrons are elevated to the conduction band leaving behind holes in the valence band, both of which migrate freely throughout the crystal until they are trapped in the crystal lattice defects.^1^, ^2^, ^3^ The free electrons are captured by shallow, intermediate, or deep electron traps depending on the energy‐band levels,^1^, ^4^ whereas holes are captured at the deep hole traps or at recombination centers.^1^, ^5^ For Al_2_O_3_:C specifically, the recombination centers are attributed to oxygen vacancies and are termed as F centers.^1^, ^5^ When a radiation generated hole is trapped by a recombination center (F), it becomes a F^+^ center.

Shallow electron traps are dosimetric traps that are shallow in the band gap, unstable in ambient temperature environments, and will release their trapped charge within a matter of minutes following exposure to ionizing radiation, in the absence of optical stimulation.^1^, ^4^ Deep electron traps are classified as a type of dosimetric trap that is filled when the OSLD accumulates a sufficiently high radiation dose and are attributed to changes in the dosimeter’s sensitivity as well as its observed supralinearity.^1^, ^4^, ^6^ Deep traps are problematic for accurate dosimetry; once charge carriers are captured by these traps, they cannot be photoionized and are immobilized for a relatively long period of time in the absence of thermal annealing at very high temperatures^2^ or short wavelength stimulation.^7^ Intermediate electron traps are characterized as the main electron traps that are energetically stable enough to retain their charge carriers at ambient temperatures but can be ionized by optical stimulation and thermal stimulation.^3^, ^8^ When the crystal is heated or optically stimulated, some electrons from the main electron traps are released into the conduction band that recombine with the ionized oxygen vacancy (F^+^ center). This results in an excited F center (F*) that decays to the ground state through the emission of a 420‐nm wavelength photon,^1^, ^8^ as demonstrated in the following equation:

(1) $$F^{+}+e^{‐}\to F^{*}\to F+hv.$$

This light is read out as OSL signal that is used in dose determination.

OSLDs, nanoDots in particular, can be reused by first clearing the stored signal in the dosimeter through the method of optical bleaching. Optical bleaching of OSLDs can be accomplished with a variety of light sources some of which include fluorescent lamps, halogen lamps, UV lamps, and light‐emitting diodes (LEDs).^9^ The advantage to optically bleaching OSLDs is that one can use a commercially available light source to clear the residual signal stored in the dosimeter, without physically damaging the dosimeter. A crucial disadvantage to optically bleaching OSLDs is its inability to delocalize electrons stored in the deep dosimetric traps^2^, ^4^, ^5^, ^9^ and thereby restore the dosimeter's original sensitivity.^3^ Adequate depletion of deep traps stored in Al_2_O_3_:C OSLDs require high‐temperature thermal annealing (900°C for 15 min), which is not suitable for nanoDots in particular due to the potential risk of melting the plastic container housing the Al_2_O_3_:C disk.^3^, ^10^ Previous works investigating the behavior of nanoDots have recommended or used blue LEDs instead of alternatives such as white or green LEDs for rapid clearance of the signal stored in OSLDs through optical bleaching^9^, ^11^ but have not accounted for nanoDots irradiated to a sufficiently high‐dose range where supralinearity was reportedly observed (>1 Gy).^4^, ^5^ Optical bleaching using a light source containing blue/UV wavelengths may be problematic regarding the reuse of OSL nanoDots given the observation that UV and blue light stimulation was reported to induce the phototransfer of charge carriers from deep dosimetric traps, via the conduction band, to shallower dosimetric traps in various samples of Al_2_O_3_:C.^3^, ^4^, ^10^, ^12^ The use of a long‐pass filter has been recommended from previous works^4^, ^7^ to optically bleach the OSLD while preventing the phototransfer of deep trap charge carriers but may require prohibitively long durations for complete optical bleaching, on the order of several hundreds or thousands of minutes depending on the accumulated dose history of the dosimeter and the irradiance of the bleaching source. This study seeks to expand on prior studies that were referenced by demonstrating that the regeneration of signal in OSLDs is caused by the exposure of the Al_2_O_3_:C disk to blue light, which may last for weeks following their optical bleaching, and to investigate the energy dependence of this phenomenon in nanoDots with prior irradiation history to 6 MV and 220 kV x rays. From this study we observed that the regeneration of signal in optically bleached nanoDot exhibit an accumulated dose, energy, and time dependence and caution the user against reusing OSL nanoDots with prior irradiation history at clinically relevant doses.

## 2 MATERIALS AND METHODS

**Fig. 1:**
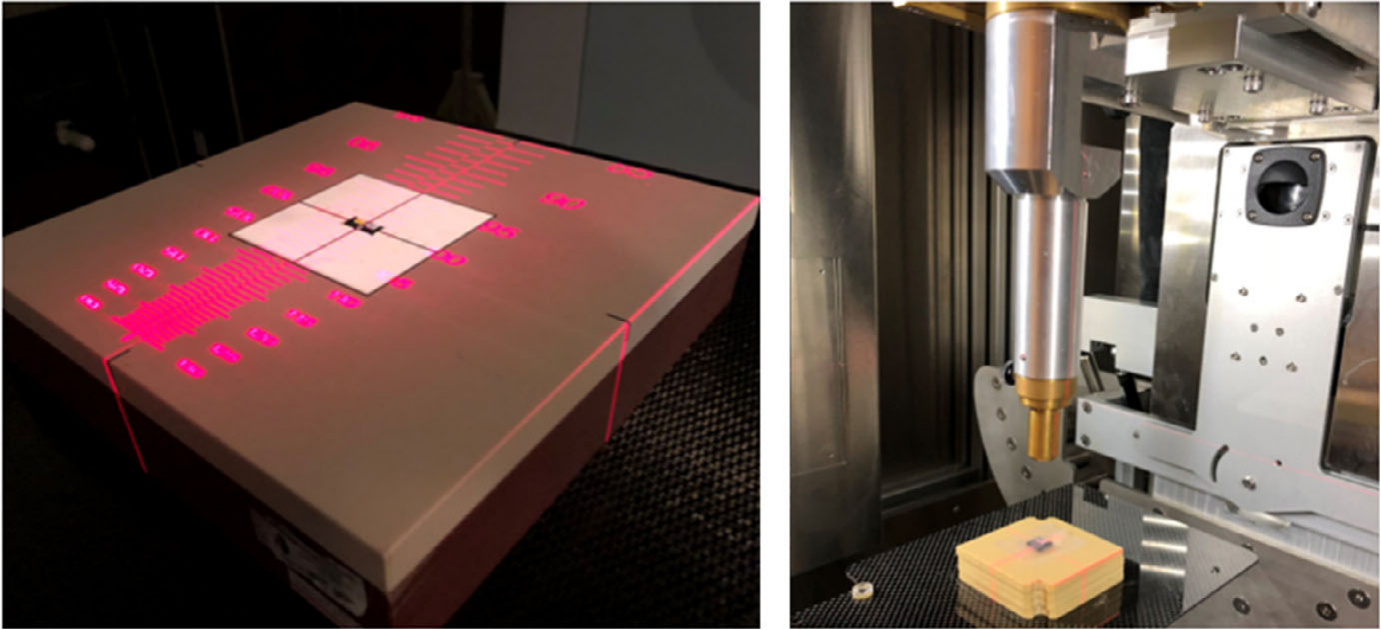
Experimental setup of optically stimulated luminescent dosimeters irradiation using 6 MV (left) and 220 kVp (right) x rays.

Thirty‐three new, unirradiated OSL dosimeters, each consisting of a 4 mm Al_2_O_3_:C disk housed in a 1 cm × 1 cm × 0.2 cm plastic casing, or nanoDots (nanoDot, Landauer, Inc., 2 Science Road, Glenwood, IL 60425‐1586, http://www.landauerinc.com/), were used for this experiment to explore issues relating to their response following exposure to ionizing radiation. The nanoDots used in this experiment were brand new, had no prior exposure history, and were each read out prior to their irradiation. The nanoDots were placed at 100 cm source‐to‐axis distance (SAD) on a 30 cm × 30 cm × 5 cm solid water phantom, as depicted in Fig. 1, with a 1.5‐cm flexible slab of solid water buildup. Irradiation of OSLDs was performed using 6 MV photons and a 4 cm × 4 cm field size. The 6 MV photons were generated from a clinical linear accelerator (ARTISTE, Siemens Medical Solutions USA, Malvern, PA, USA) that had a calibrated dose rate of 300 cGy/min at d_max_. Thirty minutes following their irradiation, the dosimeters were read out five times each using a commercially available dosimetry reader (microSTARii; Landauer, Inc., Glenwood, IL, USA) to ascertain the mean signal stored in each nanoDot. The coefficient of variation for each of these five readings was below 1.0%. A wait time following the irradiation of OSL nanoDots is necessary for accurate measurements due to the rapid signal loss observed in OSLDs when measured within the first 8 min postirradiation due to the depopulation of charge carriers in the shallow traps. The wait time between irradiation and readout for OSL nanoDots was selected based on the recommendations from AAPM TG‐191, which is a minimum of 10 min postirradiation.^13^ Daily routine quality control (QC) tests were performed, prior to use, to evaluate the reader stability. The reader’s intrinsic stability test ensures that the intrinsic measurements performed in the reader for the photomultiplier tube (PMT) and the photodiode fall within the upper and lower control limit thresholds recommended by the manufacturer and that the measured dark counts fall below the manufacturer’s upper limit of 20 PMT counts. The reading reproducibility test verifies that the reader is operating reliably for dosimetry measurements. It involves ten repeated readings of a high‐dose consistency nanoDot provided by the manufacturer, with a successful test reading resulting in a coefficient of variation of <1.0%. NanoDot readings were performed in this work only when the reader passed its daily QC test.

**Fig. 2:**
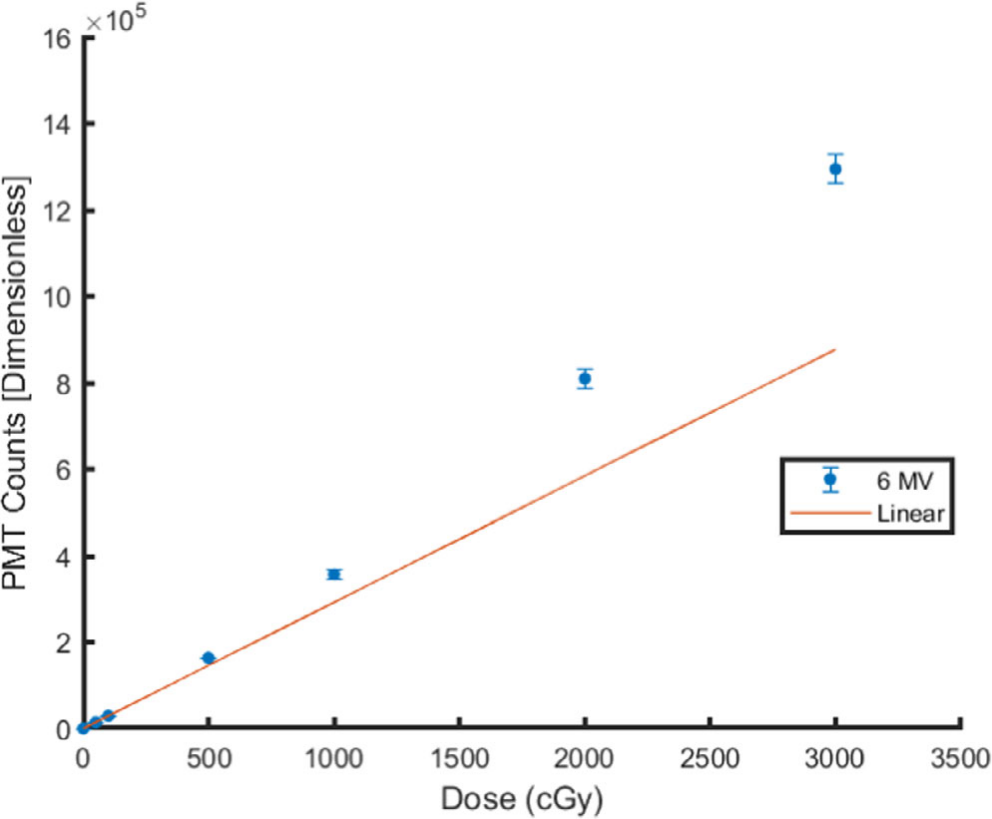
Dose Response for optically stimulated luminescent nanoDots irradiated using 6 MV photons compared to linear fit. Error bars represent 1 standard deviation from three optically stimulated luminescent dosimeters.

Twenty‐four nanoDots were utilized and examined to explore the effects of the phototransfer of charge carriers from deep traps in OSL nanoDots with prior irradiation history from 6 MV photons at different doses. The OSL nanoDots were separated into groups of three, with eight groups total, and were irradiated using 6 MV photons. The dosimeters in their respective groups were irradiated to 5, 10, 50, 100, 500, 1000, 2000, and 3000 cGy. Thirty minutes following their irradiation, the mean signal taken from five subsequent readings of the dosimeters was acquired. These readings were used to create the dose response curve in Fig. 2, comparing the measured signal in units of PMT counts to the dose delivered to the dosimeter. The dosimeters were then optically bleached using blue light to clear out the intermediate electron traps and to optically stimulate the transfer of charge carriers from the deep electron traps to shallower electron traps. These dosimeters were exposed to blue light for a period of 1 h using a standard 20 W waterproof flood lamp that contained one blue light‐emitting diode (LED) with a luminous flux of 1800 lumens (LED RGB Flood Light; Mos lighting technology Co., Ltd, Zhongshan, China) as reported by the manufacturer’s specifications. Afterwards, the OSL nanoDots were measured again to establish the baseline signal of each recently bleached nanoDot, in their respective groups, that can be used as a comparison for subsequent measurements performed and to observe any changes in signal attributed to the phototransfer of charge carriers from the deep dosimetric traps. A series of readings were performed on the optically bleached dosimeters using the reader in strong‐beam mode over a period of 27 days. The strong‐beam mode of illumination was automatically selected on the microSTARii reader during the readout process, which involves the reader LED operating at a high beam intensity to achieve better stimulation efficiency and statistical accuracy when interrogating nanoDots with low signal (primarily for low dose applications).

To compare the phototransfer of charges in OSL nanoDots irradiated to high doses and at different energies, three nanoDots were irradiated to a dose of 1000 cGy, three nanoDots were irradiated to a dose of 2000 cGy, and another three nanoDots were irradiated to a dose of 3000 cGy using 220 kV x rays generated from a small animal image‐guided irradiator (SARRP, Xstrahl Ltd., Camberley, UK) as depicted in Fig. 1. Thirty minutes following their irradiation, the OSL nanoDots were read out five times each to acquire the mean signal stored in each nanoDot postirradiation. The OSL nanoDots were then optically bleached for a period of 1 h using the same bleaching procedures mentioned previously. Following their optical bleaching, these nanoDots were measured over a 27‐day period to track changes in their measured signal over time.

## 3 RESULTS

Plot of the response of OSL nanoDots as a function of delivered dose from 6 MV photons is shown in Fig. 2. A linear response for comparison is also depicted in Fig. 2, which was derived by using the slope of the trendline developed from the origin to the 100 cGy response. The error bars represent one standard deviation from the measurement of three OSLDs at each dose value. The coefficient of variation or relative standard deviation for each of these measured values was within 3.0%.

**Fig. 3:**
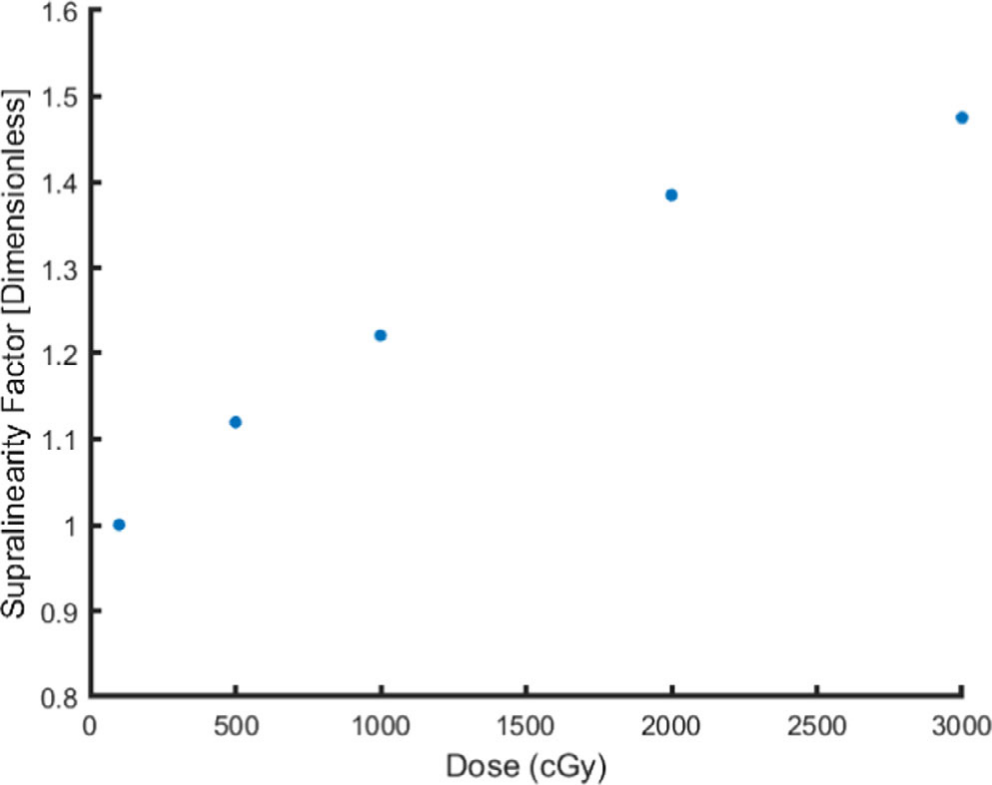
Supralinearity factor for nanoDots exposed to 100–3000 cGy using 6 MV photons.

Figure 3 plots the supralinearity factor. The supralinearity factor was defined as the dose response at delivered doses ranging from 100 to 3000 cGy, in units of PMT counts/cGy, normalized to the dose response at 100 cGy, which was measured to be approximately 293 PMT counts/cGy. There was an observed deviation (>10%) from linearity at delivered doses greater than and equal to 500 cGy, which is consistent with the observations made in previous works involving the irradiation of OSL nanoDots using 6 MV photons.^1^, ^7^

**Fig. 4:**
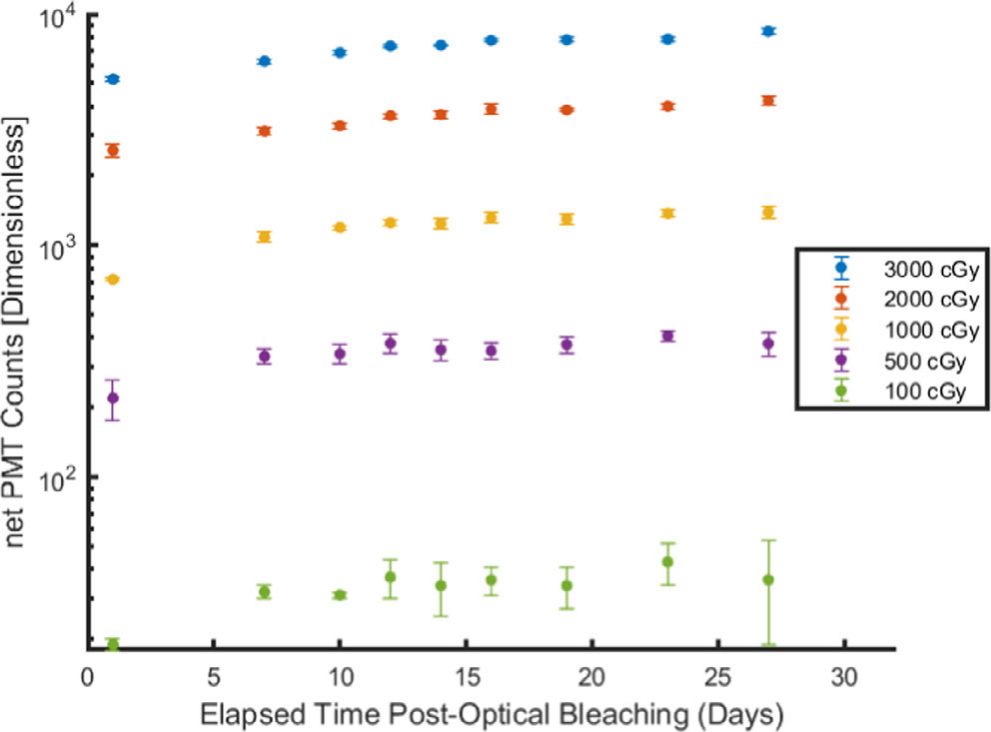
Increase in measured signal observed in optically bleached nanoDots, with prior irradiation history to 6 MV photons, due to the phototransfer of charge carriers from the deep traps to shallower traps. Error bars represent 1 standard deviation from three optically stimulated luminescent dosimeters.

Figure 4 tracks the measured growth in signal observed in optically bleached OSL nanoDots with prior exposure to 6 MV photons. The error bars plotted in Fig. 4 represent one standard deviation from the signal in three nanoDots measured at each time point. The coefficient of variation for optically bleached nanoDots with a prior history of irradiation to 500 cGy or more were within 10%. It was observed that nanoDots with a prior history of irradiation to 500 cGy or more exhibit a growth in signal of 200 PMT counts or more when measured within the first 24 h following their optical bleaching to a blue light source for a period of 1 h, while nanoDots with a prior irradiation history of 100 cGy or less exhibit a negligible change in signal of <20 PMT counts when measured within the first 24 h following their optical bleaching.

**Fig. 5:**
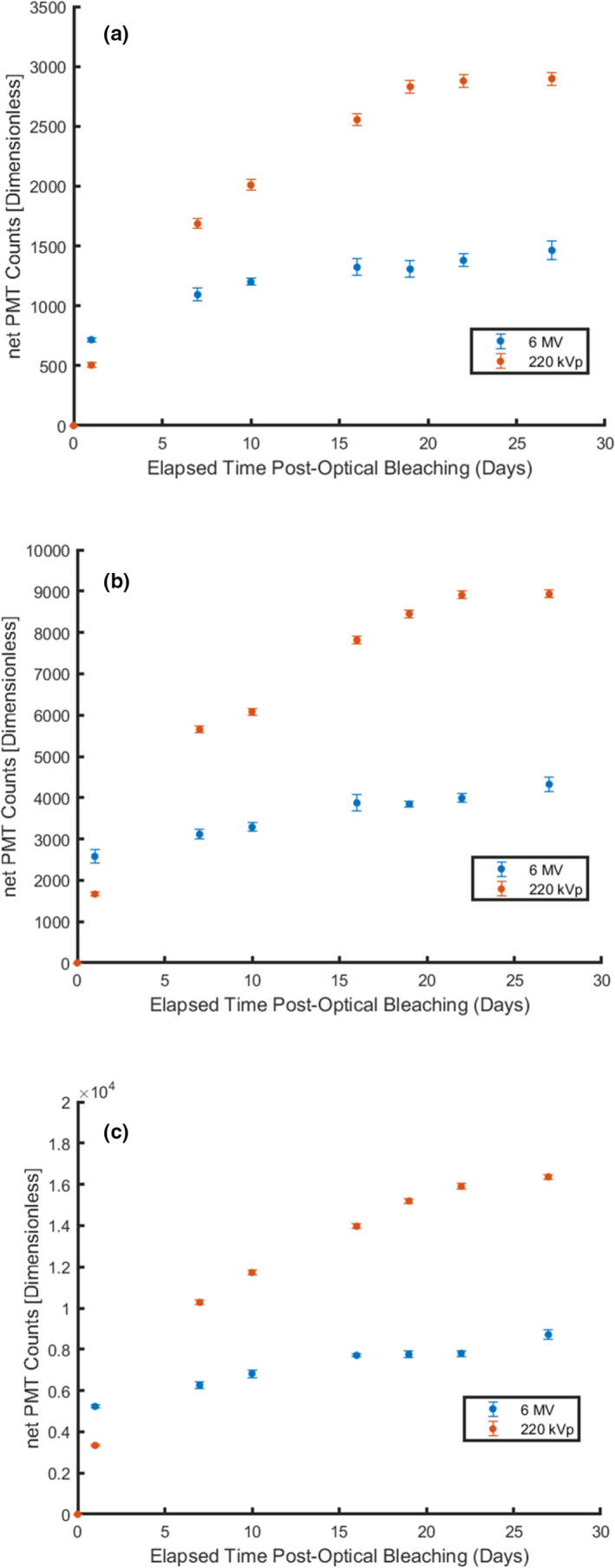
Comparison of the increase in signal, due to the phototransfer of charge carriers, observed in optically bleached nanoDots with prior irradiation history of 1000 cGy (a), 2000 cGy (b), and 3000 cGy (c) to 6 MV and 220 kVp photons. Error bars represent 1 standard deviation from three optically stimulated luminescent dosimeters.

Figure 5 and Tables 1, 2 compare the measured signal growth observed in nanoDots with prior exposure to 1000, 2000, and 3000 cGy from 6 MV and 220 kV photons. The error bars in Fig. 5 represent one standard deviation in the measured signal from three nanoDots at each time point. The coefficient of variation at each measured time point in Fig. 5 is within 5.0% for nanoDots with prior irradiation history from 6 MV photons while the coefficient of variation is within 2.0% for nanoDots with prior irradiation history from 220 kV photons. Within the first 24 h of measurement, the signal measured in OSL nanoDots with prior history from 6 MV photons is about a factor of 1.40–1.57 greater than the signal measured in OSLDs with corresponding prior dose history from 220 kV photons. However, when measured at the 7th day postbleaching, the measured growth in signal in OSL nanoDots with prior irradiation history from 220 kV photons exceeded those with prior history from 6 MV photons by more than a factor of 1.50. By the 27th day, the measured increase in signal in OSL nanoDots with prior history of irradiation from 220 kV photons was approximately twice that observed in OSL nanoDots with prior irradiation history using 6 MV photons: going as high as 16 300 PMT counts in optically bleached nanoDots with a prior irradiation history of 3000 cGy from 220 kV photons.

**Table 1:** Measured photomultiplier tube counts in bleached optically stimulated luminescent dosimeters with prior irradiation history using 6 MV x‐rays.

Elapsed Time Post‐Optical Bleaching (Days)	3000 cGy	2000 cGy	1000 cGy
0	0	0	0
1	5229	2578	717
7	6261	3114	1094
10	6818	3288	1199
16	7705	3846	1306
19	7745	3874	1322
22	7801	3989	1379
27	8710	4324	1463

**Table 2:** Measured photomultiplier tube counts in bleached optically stimulated luminescent dosimeters with prior irradiation history using 220 kVp x‐rays.

Elapsed Time Post‐Optical Bleaching (Days)	3000 cGy	2000 cGy	1000 cGy
0	0	0	0
1	3335	1665	505
7	10 277	5650	1686
10	11 724	6079	2009
16	13 967	7813	2554
19	15 192	8454	2830
22	15 903	8906	2879
27	16 363	8929	2898

## 4 DISCUSSION

Jursinic 2007 reports that supralinearity in OSL nanoDots were observed at a delivered dose exceeding 300 cGy^1^ while Omotayo et al. reports supralinearity at a delivered dose as low as 200 cGy.^7^ For nanoDots with an accumulated dose of 500 cGy, in Fig. 3, there was an observed supralinearity factor of 1.12 that increases nonlinearly to a supralinearity factor of 1.48 for nanoDots with an accumulated dose of 3000 cGy. This supralinear response is attributed to the filling of the deep electron traps, in which at higher doses more free electrons are generated in the Al_2_O_3_:C crystalline structure that subsequently occupy the deep electron traps and enhance the signal from optically stimulated luminescence.^5^

Jursinic 2010^5^ first documents the regeneration of signal in OSL nanoDots annealed using a cold fluorescent lamp (CFL). In comparing the measured increase in signal of optically bleached nanoDots with the same dose history of 3000 cGy at the time point of 24 h postbleaching, it was observed that the measured increase in signal from Jursinic 2010 was approximately 1000 PMT counts^5^ while the measured increase in signal from this work was 5200 PMT counts as shown in Fig. 4. The fivefold difference in the rate of signal regeneration between the results presented in this work and those from Jursinic 2010 for optical bleached OSLDs may be attributed to the experimental differences in the type of light source used as well as the amount of time spent bleaching the dosimeters. The regeneration of signal in optically bleached OSL nanoDots is attributed to the phototransfer mechanism in which exposure of Al_2_O_3_:C to blue light stimulates the migration of trapped charged carriers from the deep dosimetric traps to shallower dosimetric traps.^4^, ^10^ As noted by Colyott et al. 1997, the most effective wavelengths in stimulating the phototransfer of charge carriers for α‐Al_2_O_3_:C are in the short visible light to ultraviolet light range, with peak maxima observed at 280, 340, and 420 nm.^14^ Over a period of 27 days, the signal regeneration observed in OSL nanoDots reached as high a value as 8710 PMT counts for optically bleached nanoDots with a prior irradiation history of 3000 cGy and as low a value as 408 PMT counts for optically bleached nanoDots with prior irradiation history of 500 cGy. This demonstrates that OSL nanoDots with a larger accumulated dose history have a greater reservoir of electrons captured in deep dosimetric traps available to undergo migration to shallower traps following their optical exposure to blue light and that the migration of charge carriers may last several weeks following their exposure to light containing blue wavelengths. Because exposure of OSL nanoDots to blue/UV light sources was observed to induce the migration of charge carriers from the deep dosimetric traps to shallower traps, further investigation is warranted to ascertaining the possibility of completely emptying the deep dosimetric traps of stored charge carriers thereby resetting the dosimeter’s sensitivity when using a light source containing blue/UV wavelengths, whether the rate of signal regeneration is dependent on the amount of time spent bleaching the dosimeters using blue light, and the amount of time required for the regeneration of signal postbleaching to cease.

Because the filling of deep electron traps not only affects the measured nanoDot signal after their optical bleaching but also their sensitivity,^1^, ^15^ which was reported to be dependent on the bleaching time, accumulated dose, and wavelength of the bleaching source,^7^ it is recommended that OSL nanoDots not be reused with a prior irradiation history exceeding 500 cGy to minimize any further uncertainties in dosimetric accuracy. For OSL nanoDots with a prior history of 100 cGy or less, the regeneration of signal due to the phototransfer of charge carriers was observed to be no >50 PMT counts when measured over a 27‐day period, which is only substantial when reusing OSL nanoDots for very low dose exposures, on the order of several mGy.^11^ To minimize the effects that the filling of deep electron traps have on the clinical use of OSLDs, Park et al. recommends that the user first irradiate brand new OSLDs with a sufficiently high dose (>5 kGy) to fully occupy the deep dosimetric and deep hole traps, to subsequently optically bleach the dosimeter using a light source with a wavelength exceeding 495 nm to clear the intermediate dosimetric traps without inducing the phototransfer of charge carriers from the deep traps, and then to recharacterize the sensitivity of the dosimeter prior to use in clinical applications.^4^ Although this methodology may be more rigorous than what was recommended in this work, it remains impractical for physicists to implement in clinic without the use of a high activity source such as ^60^Co or ^192^Ir.^5^ For instance, the calibrated dose rate for the linear accelerator used in this work was 300 cGy/min at d_max_. The minimum amount of time required to deliver 5 kGy to a dosimeter at this dose rate would exceed 1600 min, which is not acceptable as this would occupy a single linear accelerator used in clinic for more than a day and may damage the x‐ray tube and tungsten target of the linear accelerator.

A recent work by Zhuang and Olch^16^ has suggested that OSL nanoDots may be reused with an accumulated dose of up to 7000 cGy by bleaching the nanoDots following their use and then recharacterizing the nanoDot sensitivity prior to their reuse. In their work, the authors have demonstrated that the measured OSL nanoDot sensitivities change with accumulated dose and that determination of their sensitivities can be expedited through the use of a fitting curve to predict nanoDot sensitivities from subsequent uses. The authors in this work did not specify the wavelength of the bleaching source used in their work, but if the light source used did not have a long‐pass filter to attenuate blue wavelengths then their methodology and recommendations would be inappropriate. This is because the reused nanoDot referenced in their work accumulates free electrons that are captured in the deep dosimetric traps following each irradiation cycle that are not completely delocalized after each bleaching cycle. Should the user optically bleach the nanoDot without a long‐pass filter to attenuate blue wavelengths, the nanoDots will exhibit a regeneration in their measured residual signal that may last for days, if not weeks, postbleaching depending on the accumulated dose in the dosimeter prior to optical bleaching and the amount of time the user had waited prior to reusing the dosimeter postbleaching, as demonstrated in Fig. 4. This growth in signal may in turn inflate the number of PMT counts measured in the reused dosimeter and thus obscure the dose delivered to the patient at the point that the reused nanoDot was situated.

The results from Fig. 5 and Tables 1, 2 indicate that the phototransfer of charge carriers from deep dosimetric traps not only have an accumulated dose and time dependence, but also an energy dependence as examined in the differences in measured signal of OSL nanoDots with prior irradiation history from MV and kV photons. The differences in measured signal growth, with regards to magnitude and growth rate, observed in optically bleached OSL nanoDots with prior irradiation history to MV and kV x rays may in part be attributed to the over‐response in Al_2_O_3_ at low energies due to its greater effective atomic number (Z_eff_),^17^ relative to water, thereby leading to a greater incidence of photoelectric interactions in the nanoDot.^18^ A hypothesis as to why this may be the case can be attributed to differences in photon interactions and photon cross sections in Al_2_O_3_ at kV and MV energies. At energies ranging from 1 to 200 keV, the predominant photon interactions in Al_2_O_3_ are from photoelectric interactions, based on the list of photon cross sections provided from NIST.^19^ At MeV energies, ranging from 0.5 to 6.0 MeV, the photoelectric cross section in Al_2_O_3_ becomes several orders of magnitude lower than their values at 1–200 keV energies while Compton scattering interactions become more prominent relative to photoelectric interactions. At kV energies, greater photoelectric interactions in the nanoDot per unit dose will generate greater quantities of free electrons to be captured by the shallow, intermediate, and deep dosimetric traps relative to nanoDots exposed to the same dose but from photons in the MV energy range that experience predominantly Compton scattering interactions. This may in turn allow for more electrons to be captured in the deep dosimetric traps that will migrate to shallower dosimetric traps following their optical exposure to blue light. This hypothesis answers why the magnitude in the measured regeneration of signal may be different but fails to answer why the rate of signal regeneration is different for OSL nanoDots exposed to kV and MV energies. Although the exact mechanisms remain unclear for now, further investigation is warranted in future studies of OSL nanoDots.

## 5 CONCLUSION

This study examined the dosimetric characteristics of OSLD nanoDots irradiated to absorbed doses that are clinically relevant in radiation therapy. In this study, OSLDs were observed to behave supralinearly at absorbed doses above several hundred cGy due to the filling of deep electron traps. When optically bleaching OSLDs with prior irradiation history using blue light, it is important to consider the phototransfer of charges that may arise from the movement of electrons from the deep dosimetric traps that is dependent on the history of the dosimeter as a function of dose, time measured postbleaching, and energy.
